# Functional kinome profiling reveals brain protein kinase signaling pathways and gene networks altered by acute voluntary exercise in rats

**DOI:** 10.1371/journal.pone.0321596

**Published:** 2025-04-15

**Authors:** Chia-Ming Lee, Jennifer Nguyen, Brock Pope, Ali Sajid Imami, V. William George Ryan, Smita Sahay, Victoria Mathis, Priyanka Pulvender, Hunter Michael Eby, Taylen Arvay, Khaled Alganem, Lauren Wegman-Points, Robert McCullunsmith, Li-Lian Yuan

**Affiliations:** 1 Department of Physiology and Pharmacology, College of Osteopathic Medicine, Des Moines University, Des Moines, Iowa, United States of America; 2 Department of Neurosciences and Psychiatry, College of Medicine and Life Sciences, University of Toledo, Toledo, Ohio, United States of America; 3 ProMedica, Neurosciences Institute, Toledo, Ohio, United States of America; UTMB: The University of Texas Medical Branch at Galveston, UNITED STATES OF AMERICA

## Abstract

Regular exercise confers numerous physical and mental health benefits, yet individual variability in exercise participation and outcomes is still poorly understood. Uncovering the neurobiological mechanisms governing exercise behavior is essential for promoting physical activity and developing targeted interventions for related disorders. While genetic studies have provided insights, they often cannot account for protein-level alterations, such as changes in kinase activity. Here, we employ protein kinase activity profiling to delineate brain protein kinase activity and signaling networks modulated by acute voluntary exercise in rats. Focusing on the dorsal striatum, which governs voluntary exercise, as well as the hippocampus, which is susceptible to modulation by physical activity, we aim to understand the molecular basis of exercise behavior. Utilizing high throughput kinome array profiling and advanced pathway analyses, we identified protein kinase signaling pathways implicated in regulating voluntary exercise. Pathway analysis using Gene Ontology (GO) revealed significant alterations in 155 GO terms in the dorsal striatum and 206 GO terms in the hippocampus. Changes in kinase activity were observed in the striatum and hippocampus between the exercise (voluntary wheel running, VWR) and sedentary control rats. In both regions, global serine-threonine kinase (STK) activity was decreased, while global phospho-tyrosine kinase (PTK) activity was increased in VWR rats compared to control rats. We also identified specific kinases altered in VWR rats, including the IKappaB Kinase (IKK) and protein kinase delta (PKD) families. C-terminal src Kinase (CSK), epidermal growth factor (EGFR), and vascular endothelial growth factor receptor (VEGFR) tyrosine kinase were also enriched. These findings suggest regional heterogeneity of kinase activity following voluntary exercise, emphasizing potential molecular mechanisms underlying exercise behavior. This exploratory study lays the groundwork for future investigations into the causality of variations in exercise outcomes among individuals and different sexes, as well as the development of targeted interventions to promote physical activity and combat associated chronic diseases.

## Introduction

Regular exercise exerts beneficial effects on physical and mental health. Exercise, especially when voluntary, promotes cardiovascular fitness, reduces morbidity with chronic diseases, and promotes mental wellness [1–3]. Conversely, a sedentary lifestyle, characterized by insufficient physical activity, poses deleterious effects on health and fitness, leading to amplified chronic inflammation and increasing the risk of cardiovascular, metabolic, neoplastic, and psychiatric diseases [4,5]. Despite these benefits, participation levels and performance outcomes in humans show significant heterogeneity among different individuals [3,6].

Previous studies have established that physical activity may be genetically regulated, and a host of genetic factors have been identified from animal models recapitulating individual differences, including high vs. low activity lines of inbred mice [7,8], selective breeding of high voluntary wheel running (VWR) mice [9], and high‐capacity vs. low‐capacity runners [10]. However, genetic alterations do not always reliably translate to meaningful alterations on the gene expression level [11], highlighting the necessity of research focusing on functional proteomic outcomes. Indeed, a host of molecular events induced by exercise have been identified, including neurotransmitters, metabolites, and trophic factors, most of which support the systemic beneficial effects of exercise [12,13].

Although several hypotheses exist regarding proteomic alterations regulating exercise behavior [12,14–16], current research has focused on discrete signaling molecules or a limited subset of pathways with mixed results. A recent major advance in our understanding of exercise signaling came from a global analysis of phosphorylation in human skeletal muscle following exercise. This unbiased phospho-proteomic approach successfully identified exercise-regulated kinases and their substrates, providing a detailed map of the exercise signaling network in muscle [17]. However, this study does not address the central neurobiological mechanisms involved. To visualize broader signaling networks involved in exercise behavior, further characterization of functional proteomic outcomes, including changes in kinase activity is needed. These results motivated the present investigation, focusing on protein kinases and protein phosphorylation networks in response to exercise in the dorsal striatum, a key brain region involved in goal-directed behavior and movement initiation [18,19], and the hippocampus, the most reported area to undergo alterations by exercise [13,20].

Voluntary exercise behavior of rodents, modeled by voluntary wheel running (VWR), is a well-established analogous model to study human exercise behavior and its heterogeneity [21]. Notably, acute VWR provides a controlled and reproducible model to examine immediate physiological responses to physical activity. By studying acute exercise, we may capture short-term changes in molecular signaling pathways without the confounding effects of chronic adaptations. In addition, acute exercise allows for investigating initial molecular events that may trigger downstream physiological responses and adaptations observed in chronic exercise.

In the present study, we utilized a high-throughput screening platform to profile protein kinase activity in response to acute exercise within subregions of the dorsal striatum and the hippocampus in rodents. Pathway and gene network analyses were performed to identify key kinases and signaling pathways that form a pro-exercise signaling network that may drive voluntary exercise behavior.

## Materials and methods

### Animals

6~8-week-old male Sprague Dawley rats were obtained from Charles River Laboratories (Wilmington, MA). Upon arrival, rats were acclimated in a temperature- (22 °C) and light- (12/12 h dark/light) controlled animal facility for at least one week before experiments. The rats had free access to standard laboratory rat chow and drinking water. Experimental procedures were conducted in strict adherence to the National Institutes of Health Guide for the Care and Use of Laboratory Animals of the National Research Council of the (U.S.) National Academies and were approved by the Des Moines University Institutional Animal Care and Use Committee.

### Voluntary wheel running (VWR)

After a 7-day acclimation period, rats were housed individually in polycarbonate living chambers (40.64 × 50.80 × 20.96 cm) equipped with stainless steel lids and running wheels with a circumference of 1.10 meters (Scurry Rat Activity Wheel with Living Chamber, Lafayette Instrument). Scurry Rat Activity Counters were mounted to the wheels and connected to the Scurry Interface for Animal Activity. The interface was connected to a computer, and the use of each running wheel was reported in real-time and stored in the Scurry Activity Monitoring Software (Lafayette Instrument). The cumulative distance traveled on each running wheel was recorded every 24 hours throughout the duration of each experiment.

### Brain tissue collection and processing

For tissue collection, rats were lightly anesthetized with isoflurane and euthanized by rapid decapitation using a rodent guillotine. Brain tissue was collected on ice and samples were stored at -80°C until processing. The hippocampus or tissue punches from the dorsal striatum were homogenized and lysed in M-PER (mammalian protein extraction reagent, M-PER) (Thermo Scientific) with protease inhibitor cocktail and phosphatase inhibitor cocktail. Samples were centrifuged (14,000 rpm, 10 min, 4 °C), and the supernatants were collected and assayed for total protein concentration (Pierce BCA Protein Assay Kit, ThermoFisher).

### Kinome array profiling via the PamGene platform

The kinome activity profiling platform PamStation12 was used to measure kinome activity using the STK and PTK PamChip (PamGene International) as previously described [22–26]. PamChips are available to interrogate two distinct subsets of the kinome: the phospho tyrosine kinase (PTK) Chip and the serine/threonine kinase (STK) Chip. Each PamChip has four wells, allowing four samples to be loaded on a single chip, and the Pamstation12 can accommodate three chips of either STK or PTK per run. For the PTK Chip, each well contains 196 reporter peptides in a 14 × 14 grid that can be phosphorylated by tyrosine kinases, while for the STK Chip, there are 144 reporter peptides set in a 12 × 12 grid pattern. The assay is based on the PamStation12 standard protocols [22–26]. Briefly, brain homogenates were diluted to recommended concentrations (0.2 µg/µL for the STK and 1 µg/µL for the PTK) and run in triplicate using three separate PamChips. Identical protein amounts were loaded for each condition. The assay was performed by blocking each array with 2% bovine serum albumin (BSA) before 2 ug of protein of the samples, and 157 µM adenosine triphosphate (ATP), and a primary antibody mixture as a part of the two-step reaction process designed for STK PamChips. For the second step, FITC-labeled anti-phospho serine-threonine antibodies (PamGene) were added to each array. The homogenized samples, alongside the assay mix, were pumped through the wells through timed cycles to catalyze the reaction between kinases in the sample and the reporter peptides on the chip. The degree of phosphorylation per well was measured in real-time using Evolve (PamGene) kinetic image capture software. The Evolve software captures images of FITC-labeled anti-phospho antibodies binding to each phosphorylated peptide substrate for each timed cycle. Peptide spot intensity was captured across multiple exposure times (10, 20, 50, 100, 200 ms) during the post-wash phase. The BioNavigator software package (PamGene) was used to convert the captured images to numerical values based on the intensity levels. Full details for the kinome array workflow are included in the *Supplementary methods* [23–27].

### Upstream kinase analysis

To identify upstream kinases based on differences in phosphorylation of reporter peptides on the STK and PTK chips, three different software packages were used: Kinome Random Sampling Analyzer (KRSA), upstream kinase analysis (UKA), and Kinase Enrichment Analysis Version 3 (KEA3). To look at associated upstream kinase families, KRSA takes the list of differentially phosphorylated peptides and uses a random resampling approach to assign scores for each kinase family [27]. Additionally, the Upstream Kinase Analysis (UKA) tool from BioNavigator was used to look at individual upstream kinases. The default settings of the standard chip analysis protocol were used with the additional step of upstream kinase analysis. UKA reports the final score as a metric for ranking implicated kinases [28]. The kinase final score is calculated based on the specificity of the peptides mapped to the kinases and the significance of phosphorylation changes of the peptides. The KEA3 web tool was also used to perform kinase set enrichment analysis using the corresponding proteins of the top differentially phosphorylated reporter peptides as the input [29]. More details for these packages are provided in the Supplementary methods section.

### Integration of upstream kinase assignments across packages

We used the Creedenzymatic R package Version 6.1.0 to aggregate the results from these three different analytic tools [23]. The Creedenzymatic R package is a pipeline software package that combines, scores, and visualizes the results from multiple upstream kinase analytic tools (https://github.com/CogDisResLab/creedenzymatic). The Creedenzymatic package harmonizes the different metrics used in KRSA, UKA, and KEA3 with percentile rank normalization. This harmonization results in a unified percentile score for each kinase under each tool. Then, the mean and median percentile score for each kinase is calculated by averaging the normalized scores across the three analytic tools. Additionally, kinases are mapped to the official HUGO Gene Nomenclature Committee (HGNC) symbols and subfamilies, ensuring the naming convention is consistent across the four different tools [23]. More details for this package are provided in the Supplementary methods section.

### Kinase activity

Changes in the activity level of specific kinases were assessed by calculating the log2 fold change (Log2FC) in phosphorylation levels for each specific substrate. Substrates with a Log2FC greater than 0.2 were assigned a value change of +1, those with a Log2FC between -0.2 and 0.2 were assigned a value of 0, and those with a Log2FC less than -0.2 were assigned a value of -1. Subsequently, the values for all substrates associated with a particular kinase were averaged. A direction of change was determined based on whether the average value was above or below zero.

### Network-based kinome pathway analysis

We used the Kinograte R package [30], which implements an optimized version of the well-established PCSF algorithm [31], to generate an integrated protein-protein interaction (PPI) network consisting of all STK and PTK kinomic “hits” in the hippocampus (HPC) or dorsal striatum (STR). We assigned node prizes by percentile rank of mean Creedenzymatic kinome rank across each analytic tool and edge costs by inverse STRING-DB [32] interaction confidence. We then performed gene-set over-representation analysis using the resulting PPI network’s nodes as input to the Enrichr [33] web app with the 2023 Gene Ontology database [34] to identify dysregulated pathways (FDR adjusted p-value < 0.05). We visualized the resulting PPI subnetworks of these pathways in HPC or STR to show molecular interactions between kinases and algorithmically identified relevant “hidden nodes.”

### Functional interpretation of dysregulated pathways

Dysregulated pathways (FDR < 0.05) were functionally clustered and visualized using PAVER [35], a meta-clustering method for pathways. PAVER finds the most representative terms (MRTs) for hierarchically clustered pathway embeddings [36] by selecting whichever term is most cosine similar to its respective cluster’s average embedding. We generated UMAP scatter plots of individual pathways colored and shaped by the cluster they belong to and the experimental comparison (HPC or STR) they came from, respectively. We then generated heatmaps showing normalized enrichment scores (NES) of individual pathways in their identified cluster, hierarchically clustered by the average enrichment of each cluster.

### Nuclear extraction and measurement of NF-kB kinase activity

Homogenized and lysed hippocampal tissue was processed using the Nuclear Extraction Kit (Cayman Chemical, Ann Arbor, MI) according to the manufacturer’s instructions, which separates each sample into cytosolic and nuclear fractions. The protein concentration for each fraction was determined using a Bicinchoninic Acid Assay (BCA) kit (Pierce; Rockford, IL), and each nuclear extract was calibrated to equal concentrations. Using the NF-kB Transcription Factor Assay Kit (Cayman Chemical, Ann Arbor, MI) according to the manufacturer’s instructions, nuclear extracts were incubated in wells coated with consensus dsDNA sequence to which active NF-kB would bind. Serial dilutions of a positive control were also incubated concurrently. The wells were treated with ∝-NF-kB antibodies, then horseradish peroxidase reagents. Absorbances were read at 450nm, and a standard curve was built with the absorbance of the serial dilutions of the positive control. Sample absorbances were plotted on the standard curve to measure standardized NF-kB activity.

### Data collection and analysis

Data are expressed as mean ± SE. VWR output data were collected from the Scurry Activity Monitoring Software (Lafayette Instrument, Lafayette, IN). Statistical analysis and figures were completed in GraphPad Prism (Dotmatics, Boston, MA). Statistical significance was determined by Student’s t-test when two groups were compared or ANOVA and Tukey’s post hoc analysis when more than two groups were compared. To evaluate the relationship between continuous variables, simple linear regression was performed in GraphPad Prism (Dotmatics, Boston, MA) to determine the coefficient of determination (R^2^) and *P*-value. Significance was defined as *P ≤ 0.05.*

### Supplementary methods

Additional detailed information for experimental design, data collection, and analysis for our peptide array platform is available in the Supplementary information in S1 File.

## Results

### Overall workflow

**Fig 1:**
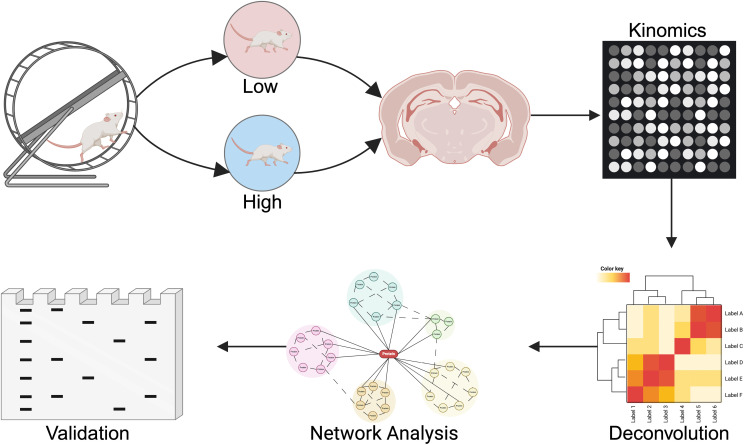
Overview of workflow. Workflow deployed to explore changes in brain mechanisms that regulate exercise behavior. The PamStation12 kinome array platform was used to profile activity in response to exercise within specific brain regions. Kinase substrate-mapping databases were used to deconvolve peptide phosphorylation patterns and determine differential kinases. Serine/threonine and tyrosine kinase activity profiles were combined using Prize Collecting Steiner Forest (PCSF) to identify the kinases and their affiliated networks and pathways. Top candidates were validated in an independent cohort of tissue samples using standard biochemical assays.

The kinome profiling process involves homogenizing samples from specific brain regions of acutely running rats and their sedentary controls, followed by kinome array profiling and bioinformatical analysis using specialized software packages (Fig 1). Biological replicates for each running condition and brain region (4 separate groups total) were pooled, aliquoted, assessed for protein content, and stored at −80C. Biological replicates for each running status and brain region were thawed, pooled, and run in technical triplicate across three STK or PTK PamChips to assess the activity of serine-threonine (STK) and tyrosine kinases (PTK), respectively. Due to failure of a chip, only two out of the three technical replicates for the STK chips were analyzed. Data analyses were performed within each chip, and log2 fold change data was averaged across technical replicates.

This study utilized two cohorts of rats. Cohort 1 included a sedentary group (N = 6) and a voluntary wheel running (VWR) group (N = 7). From each group, three rats were selected for high-throughput proteomic profiling. Similarly, Cohort 2 comprised a sedentary group (N = 6) and a VWR group (N = 13). Rats from Cohort 2 served as an independent group for molecular candidate validation.

### Individual differences revealed by an acute VWR paradigm

Our long-term goal is to understand how organisms benefit from regular exercise, such as chronic running consisting of repetitive bouts of running. We initiated our study with an acute program before delving into a chronic wheel-running paradigm. This approach allows us to establish a baseline understanding of the immediate molecular changes that occur because of the activity, which may provide valuable insights and context for interpreting the long-term effects of the chronic wheel-running paradigm.

**Fig 2:**
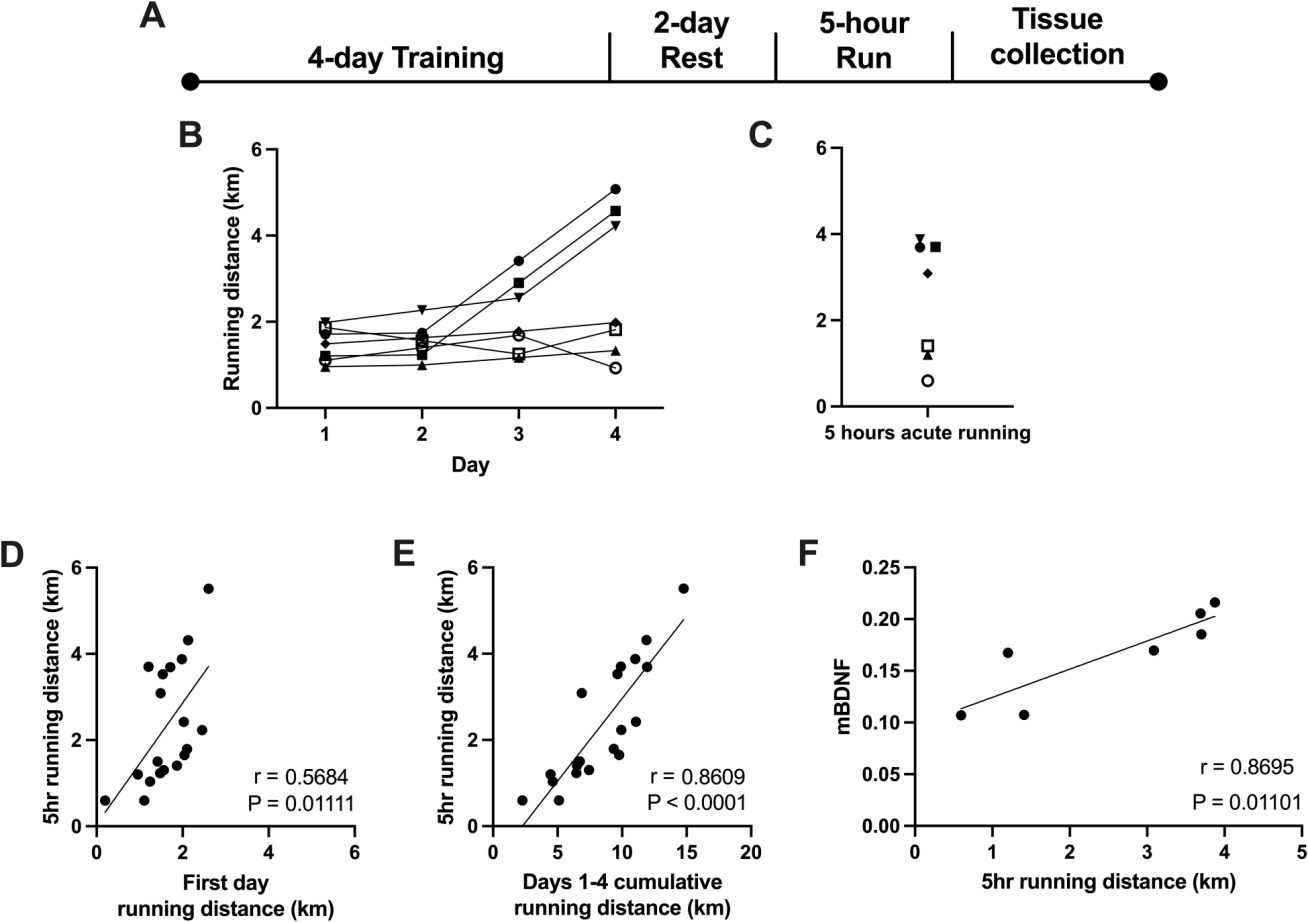
Individual differences in acute voluntary wheel running (VWR) performance. **(A)** Acute VWR experimental timeline. **(B)** Representative daily running performance of male acute VWR participants during the 4-day training period (Cohort 1, n = 7). **(C)** Acute (5hr) running performance of each participant of the Cohort 1 (n = 7) **(D)** Correlation between acute running distance and first-day running distance for each participant from Cohorts 1 and 2 (n = 20, R^2^ = 0.3585, p < 0.05). **(E)** Correlation between the cumulative running distances on days 1-4 and the acute running distance for each participant (n = 20, R^2^ = 0.7608, p < 0.05). **(F)** Correlation between acute running distance and hippocampal mBDNF (Cohort 1 n = 7, R^2^ = 0.76, p < 0.05).

We investigated acute exercise responses using an exercise program consisting of a 2-day acclimation to the locked wheels, 4 days of free access to the running wheels, followed by a 2-day rest, and then 5-hour access to wheels before tissue collection (Fig 2A). During the 4-day free access period, daily wheel running distances were recorded for each rat (representative performance of 7 rats from Cohort 1 is shown in Fig 2B). After the 4-day acclimation period and 2-day rest, rats gained access to wheels for a 5-hour period, and their individual running activity was documented (Fig 2C). This single acute session of 5-hour running, consistently taking place from 6 pm to 11 pm, was effective in exhibiting individual differences in VWR that began to manifest in the first four days of training. To determine if individual differences in running behavior persisted from day one of running, we compared first-day running distances and acute running distances. To enhance statistical power, we combined data from Cohorts 1 and 2, revealing a positive correlation (n = 20, R^2^ = 0.3585, *p* < 0.05) (Fig 2D). Similarly, we calculated cumulative running distances of the 4-day training period for each rat and compared them to their acute running distances and observed a strong positive correlation (n = 20, R^2^ = 0.7608, *p* < 0.05) (Fig 2E). Additionally, hippocampal tissue from Cohort 1 was collected immediately following the 5 hours of running and processed for western blot analysis. A positive correlation was observed between acute running distances and mature BDNF (mBDNF) expression (n = 7, R^2^ = 0.76, *p* < 0.05) (Fig 2F). This finding suggests that mBDNF is upregulated in the hippocampus by increased VWR activity.

### Global changes in serine-threonine kinase activity induced by acute VWR

Given the wide range of voluntary running activity observed within our cohort of tested rats and its positive correlation with specific molecular changes, we combined biological replicates from the top three performers of VWR to ensure the reliability of our kinome screening results.

**Fig 3:**
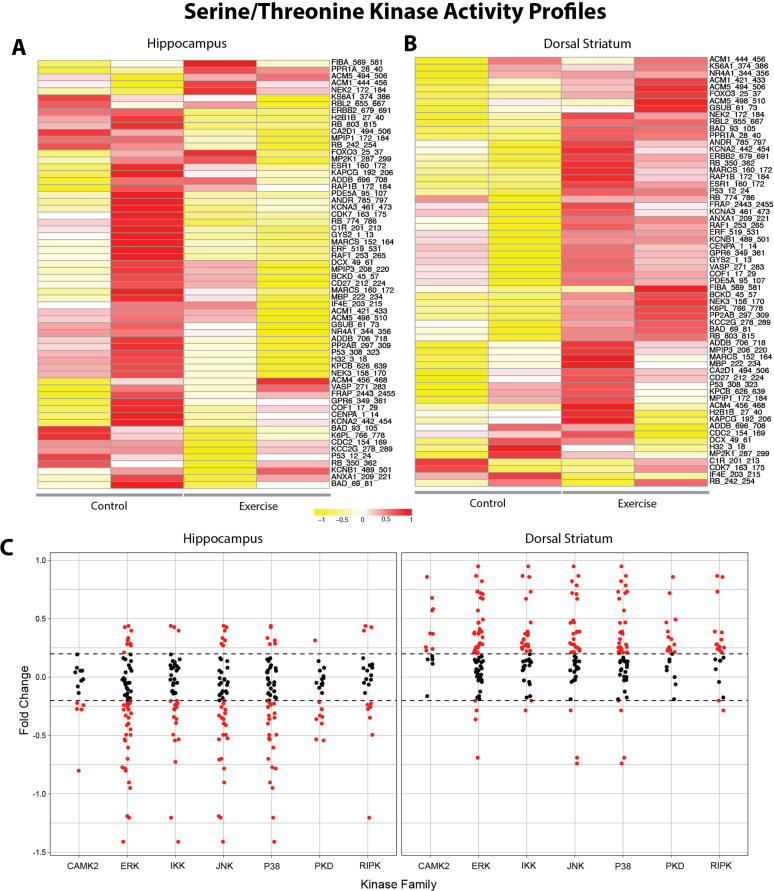
Differential kinase activity of exercised versus control rats within the striatum and hippocampus on serine/threonine kinase PamChip. **(A and** **B)** Row-normalized heatmap illustrates changes in peptide phosphorylation in control and exercised-rat hippocampus and striatum brain homogenate. Samples were assessed using a serine-threonine kinase (STK) peptide array on the PamGene Station12. The heat map scale is determined by relative signal intensity, with higher phosphorylation represented in red and lower phosphorylation in yellow for each reporter peptide on the array. **(C)** Fold-change of phosphorylated STK substrates mapped to kinase families, showing region-specific changes in exercised rats. The x-axis represents distinct kinase families, while each dot on the scatterplot corresponds to an individual reporter peptide. Dashed lines indicate a log fold-change (logFC) threshold of ±0.20, denoting biologically significant changes. Reporter peptides exhibiting logFC values beyond this threshold (|logFC| > 0.2) are highlighted in red.

Immediately following the last bout of rat wheel running, we collected brain tissue from the dorsal striatum and hippocampus. The dorsal striatum plays a pivotal role in goal-directed behavior and movement initiation, while the hippocampus is altered in response to exercise. To determine changes in protein kinase activity profiles following the last bout of wheel running, we generated global heatmaps with unsupervised hierarchical clustering using differentially phosphorylated peptides that passed quality control (QC) steps. The quality control steps included eliminating peptides with zero or undetectable signals or ones that did not have a linear increase in signal intensity over time. Notably, the hippocampus displayed 30 out of 144 reporter peptides that passed QC (Fig 3A). We observed lower average signal in the VWR samples compared to the sedentary controls, suggesting lower STK kinase activity in the hippocampus of VWR rats.

The dorsal striatum displayed 34 out of 144 reporter peptides that passed QC (Fig 3B). In contrast to the hippocampus, we observed an increase in VWR rats compared to the controls, indicating higher STK kinase activity in the dorsal striatum of VWR rats. The signal intensities of each sample in each technical replicate are depicted in Fig 3A. To identify differential kinase activity in VWR rats, we computed the log_2_-fold change (Log2FC) in phosphorylation levels for each reporter peptide mapped to each kinase family (Fig 3C). In the dorsal striatum, higher activity was observed on the STK chip for ERK, IKK, JNK, P38, PKD, and RIPK kinase families. Conversely, the same kinase families showed lower STK kinase activity in the hippocampus.

### Global changes in tyrosine kinase activity induced by acute VWR

**Fig 4:**
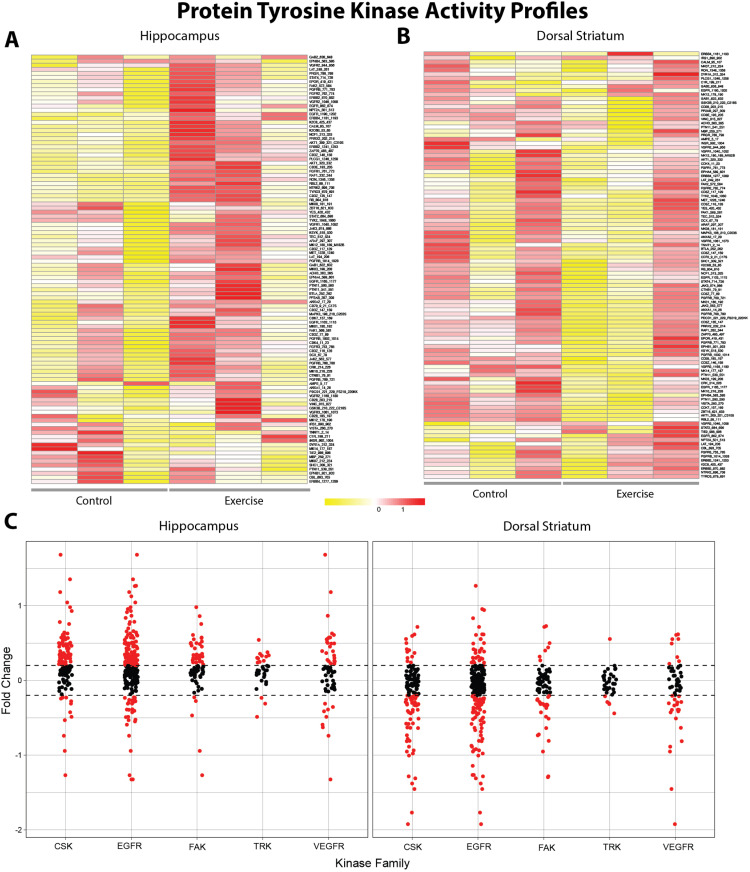
Differential kinase activity of exercised versus control rats within the striatum and hippocampus on phospho-tyrosine PamChip. **(A and** **B)** Row-normalized heatmap illustrates changes in peptide phosphorylation in control and exercised-rat hippocampus and striatum brain homogenate. Samples were assessed using the phospho-tyrosine kinase (PTK) peptide array on the PamGene Station12. The heat map scale is determined by relative signal intensity, with higher phosphorylation represented in red and lower phosphorylation in yellow for each reporter peptide on the array. **(C)** Fold-change of phosphorylated PTK substrates mapped to kinase families, showing region-specific changes in exercised rats. The x-axis represents distinct kinase families, while each dot on the scatterplot corresponds to an individual reporter peptide. Dashed lines indicate a log fold-change (logFC) threshold of ±0.20, denoting biologically significant changes. Reporter peptides exhibiting logFC values beyond this threshold (|logFC| > 0.2) are highlighted in red.

Next, we examined the changes in tyrosine kinase activity profile in pooled biological samples from the top three performers of VWR in comparison to sedentary controls in the dorsal striatum and hippocampus. We generated global heatmaps with unsupervised hierarchical clustering after quality control steps, including eliminating peptides with zero or undetectable signals or ones that did not have a linear increase in signal intensity over time. Of note, hippocampus displayed 54 out of 196 reporter peptides that passed QC (Fig 4A). We found higher averaged signal in VWR samples compared to the control samples, suggesting higher kinase activity in the hippocampus.

On the other hand, the dorsal striatum displayed 40 out of 196 reporter peptides that passed QC (Fig 4B). The decreased in averaged signal in the VWR samples compared to the control samples suggests lower activity of tyrosine kinases in the dorsal striatum. The signal intensities of each sample in each replicate are depicted in Fig 4. To identify the differential kinase activity in VWR rats, we calculated the log2 fold change (Log2FC) in phosphorylation levels for each specific substrate (Fig 4C). In the hippocampus, higher activity was observed on the PTK chip for the CSK, EGFR, and FAK kinase families. In contrast, the CSK, EGFR and FAK had lower activity in the dorsal striatum.

### Identification of Individual serine/threonine kinases altered in VWR rats

**Fig 5:**
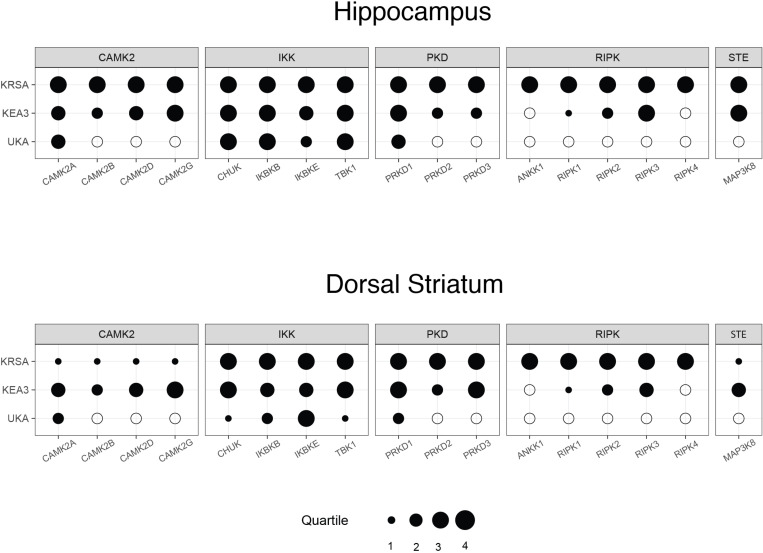
Comparison of upstream serine/threonine kinase importance for exercised rat striatum and hippocampus. Bubble plot showing the relative rank of each kinase and kinase family across three different methods of discovering upstream kinases from reporter peptides as generated from the kinome activity profile of serine/threonine kinases in rat hippocampus (**A**) and dorsal striatum **(B)**. Kinase family names appear along the top, while individual kinases belonging to those families appear along the bottom. Each row represents one method of assigning upstream kinases to kinome array output. This includes KRSA, UKA, and KEA3. Filled-in circles represent the quartile rank of the kinase within each method, and larger circles correspond to higher quartiles (taking quartile 1 to be the lowest) and, consequently, higher confidence in the involvement of that kinase. Empty circles represent kinases that are absent in the output of that method.

To identify the kinases involved in these changes in VWR rats, we performed three kinds of upstream kinase identification analyses. The input to these analyses were the reporter peptides with at least a 15% change in their activity based on fold-change calculation. This was based on our earlier approach [25] and existing literature suggesting that a 15% change in activity is enough to cause a biologically meaningful change [37–40]. The three methods utilized were Kinome Random Sampling Analyzer (KRSA), Upstream Kinase Analysis (UKA) and Kinase Enrichment Analysis (KEA3). Each of these methods returns a ranked list of kinases along with their enrichment scores. We harmonized the results from these three deconvolution techniques using the Creedenzymatic R package, which allows for direct comparisons between these three methods using a bubble plot. Our results show that the IKappaB Kinase (IKK) family is enriched in the hippocampus (Fig 5A) and striatum (Fig 5B), including the three major members CHUK (also known as IKKα), IKBKB (IKKβ), and IKBKE (IKKε). These kinases are direct regulators of the NF-κB (nuclear factor kappa-light-chain-enhancer of activated B cells) signaling pathway. In addition, TBK1 (TANK-binding kinase 1) is related to the IKK family. Furthermore, members of the Serine/threonine-protein kinase Delta (PKD) family are also significantly enriched, with PRKD1 being highly enriched across all three deconvolution methods. The Ca2+/Calmodulin-dependent protein kinase II (CAMK2) family is also highlighted, with CAMK2A being enriched across all tools.

### Identification of individual tyrosine kinases altered in VWR rats

**Fig 6:**
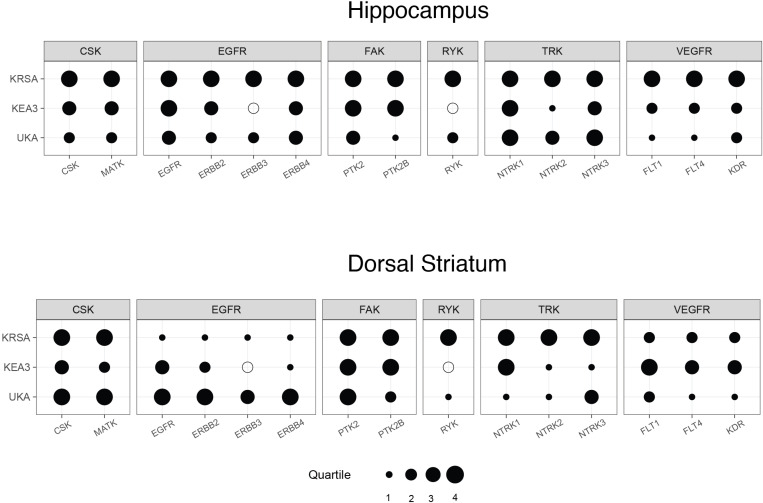
Comparison of upstream tyrosine kinase importance for exercised rat striatum and hippocampus. Bubble plot showing the relative rank of each kinase and kinase family across three different methods of discovering upstream kinases from reporter peptides as generated from the kinome activity profile of tyrosine kinases in rat hippocampus (**A**) and dorsal striatum **(B)**. Kinase family names appear along the top y-axis, while individual kinases belonging to those families appear along the bottom y-axis. Each row represents one method of assigning upstream kinases to kinome array output. This includes KRSA, UKA, and KEA3. Filled-in circles represent the quartile rank of the kinase within each method, and larger circles correspond to higher quartiles (taking quartile 1 to be the lowest) and, consequently, higher confidence in the involvement of that kinase. Empty circles represent kinases that are absent in the output of that method.

Using the same technique, we assessed the PTK dataset to identify significantly enriched tyrosine kinases. Results are summarized in the bubble plots for the hippocampus (Fig 6A) and the dorsal striatum (Fig 6B). The C-terminal Src Kinase (CSK) family of kinases, including the members CSK and Megakaryocyte-Associated Tyrosine-Protein Kinase (MATK) subfamilies, were significantly enriched across all three tools. Another important kinase family was the Epidermal Growth Factor Receptor (EGFR) and the Vascular Endothelial Growth Factor Receptor (VEGFR) families of PTKs. Related to the Receptor Tyrosine Kinase (RYK) protein, an atypical growth factor receptor kinase was also highly enriched.

### Generation of pro-exercise kinase networks

**Fig 7:**
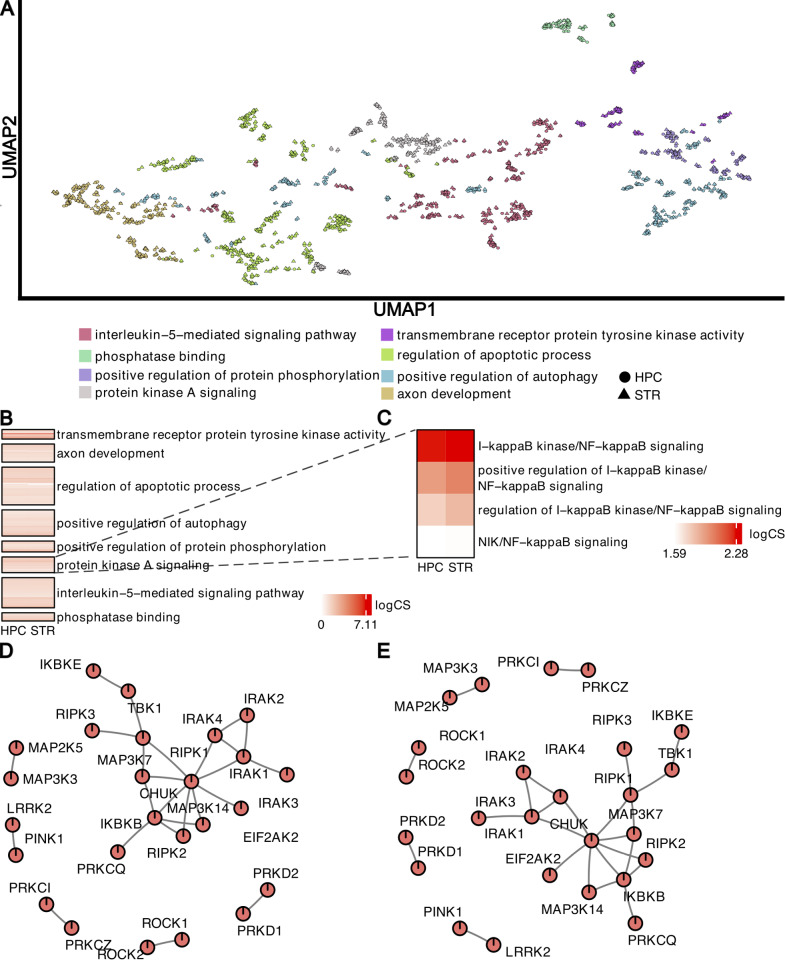
Detection of changes in molecular pathways and networks within the striatum and hippocampus of exercised rats using multi-omics integration. **(A)** PAVER analysis of dysregulated molecular pathways revealed the presence of 8 functionally distinct clusters of Gene Ontology (GO) terms within the Uniform Manifold Approximation and Projection space. Triangles on the UMAP denote striatal pathways, circles denote hippocampal pathways. **(B)** Pathway cluster heatmap showing the enrichment (log combined score) of pathways in the identified gene set clusters, arranged from most (top) to least (bottom) enriched. **(C)** Targeted heatmap cluster of protein kinase A signaling showing the enrichment of pathways in the identified gene set cluster arranged from most (top) to least(bottom) enriched. (**D and** **E**) Focal subnetworks illustrating protein-protein interactions of NF-kB pathway genes were identified in the striatum (**D**) and hippocampus **(E)**, specifically involving the IKK subunits IKKα (CHUK), IKKβ (IKBKB), and IKKε (IKBKE). Notably, CHUK serves as a central node in both subnetworks and is also known as IKKα, a potent modulator of NF-kB signaling. PAVER: Pathway Analysis Visualization with Embedding Representations; HPC: Hippocampus; STR: Striatum; log CS: log combined score.

To detect changes in kinomic molecular pathways, we performed a network-based integration and pathway analysis of our different kinome datasets. Significantly altered GO terms in dorsal striatum and hippocampus clustered into eight functional groups, including “axon development,” “regulation of apoptotic process,” and “protein kinase A signaling.” (Fig 7A–C). We investigated the enrichment of pathways in the “protein kinase A signaling” cluster, which revealed four gene sets related to positive regulation of NF-kB signaling. We then generated focal subnetworks showing protein-protein interactions (PPI) of NF-kB gene-sets in dorsal striatum or hippocampus, which identified CHUK (conserved helix-loop-helix ubiquitous kinase, also known as IKKα) as a top hub node in both dorsal striatum (Fig 7D) and hippocampus (Fig 7E). The other two subunits of the IKK complex, IKBKB (IKKβ) and IKBKE (IKKε) were also identified, highlighting the involvement of the NF-kB pathway.

### Validation of the NF-kB pathway identified from the high throughput proteomic profiling

Now that we identified a network involving the NF-kB signaling pathway for both brain regions (Fig 7D and E). While the network package software does not indicate directionality, the kinase activity data (Figs 3C and 4C) suggest that there is lower STK signaling in the hippocampus network, with higher STK signaling in the striatum, particularly for NF-kB associated kinases.

**Fig 8:**
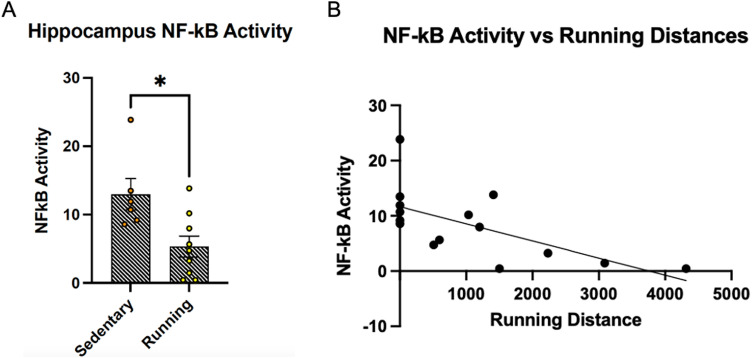
NF-kB activity is downregulated in the hippocampus of rats following acute exercise. **(A)** Group comparison of standardized NF-kB activity in the hippocampus between the sedentary control and acute voluntary wheel running group (control = 12.96 ± 2.30, n = 6; VWR = 5.33 ± 1.54, n = 9; p = 0.013). **(B)** Scatter plot of standardized hippocampal NF-kB activity in sedentary and acute VWR rats. Sedentary rats were assigned a running distance of 0 since they did not run. A line fit indicates that NF-kB activity is negatively correlated to running distance during acute exercise (R^2^ = 0.424, n = 15; p = 0.022).

To validate this finding, we directly assessed NF-kB gene expression levels in an independent cohort of tissue samples. This cohort consisted of 6 sedentary and 13 VWR rats. Brain tissue was successfully obtained from 6 sedentary and 9 VWR rats. Using a transcriptional assay, we measured NF-kB activity in the hippocampus immediately following acute exercise. Quantitative analysis revealed a significant reduction in NF-kB activity in the acute exercise group compared to sedentary controls (Sedentary control = 12.96 ± 2.30, n = 6; VWR = 5.33 ± 1.54, n = 9; p = 0.013) (Fig 8A).

Since voluntary running reduced overall NF-kB activity, we hypothesized that individual animals running more during the voluntary running period would exhibit a greater downregulation of NF-kB activity compared to less active animals. Sedentary rats, which did not run, were assigned a running distance of 0. Linear regression analysis of individual NF-kB activity versus running distance revealed a moderate negative correlation, indicating that animals running the most showed more pronounced NF-kB downregulation (R² = 0.424, n = 15; p = 0.022) (Fig 8B). When sedentary rats were excluded from the analysis, the negative correlation between NF-kB activity and running distance persisted but was no longer statistically significant (R² = 0.294, n = 9; p = 0.131) (S2 Fig in S1 File).

## Discussion

### Application of functional proteomics to exercise behavior

Despite the well-known health benefits of exercise, our understanding of individual variability in exercise outcomes remains limited. In this study, we aimed to profile exercise-induced changes in kinase activity in rats immediately after five hours of voluntary wheel running. This model revealed individual differences in exercise performance, such as running distance and time, allowing us to identify molecular alterations linked to these behavioral differences. In the dorsal striatum and hippocampus, we found significant changes in kinase activity between sedentary and VWR rats, particularly involving the IKK and PKD families. In addition, we observed enrichment in the CSK, EGFR, and VEGFR tyrosine kinase families. Previous multi-omics studies found that acute physical activity induces widespread molecular changes across various organ systems and pathways, including protein kinase activity, especially in pathways related to inflammation and metabolism [41]. Our findings are largely consistent with this prior data. While the molecules identified in our high-throughput approach come from a discovery-based pipeline, they have the potential to generate hypotheses for future causality investigations.

Moreover, our study expands the current understanding by focusing on kinome profiling, specifically in the dorsal striatum and hippocampus - brain regions critical for regulating exercise and exercise behavior [13,18–20]. The differential kinase activity observed in these regions suggests that exercise-induced molecular changes are not uniform across the brain, contributing to the diverse behavioral and cognitive outcomes associated with acute exercise. Our findings underscore the importance of considering regional brain variations and individual differences in exercise performance to fully understand the molecular mechanisms underlying exercise behavior.

### Region-specific kinome changes induced by acute running

Our findings reveal distinct region-specific changes in kinase activity induced by acute voluntary running. The dorsal striatum exhibited a significant increase in global tyrosine kinase activity, suggesting potential protein kinase targets associated with exercise-induced signaling pathways. In contrast, the hippocampus showed higher global serine/threonine kinase activity, highlighting distinct molecular responses in these brain regions. These contrasting patterns provide insight into the complex neurobiological processes underlying voluntary wheel running (VWR) and suggest that exercise regulates kinase activity in a region-specific manner, consistent with previous studies demonstrating region-specific roles of the dorsal striatum and hippocampus during exercise [42,43].

The dorsal striatum’s role in motor control and reward-related behaviors is well-established [42], and tyrosine kinases are known to regulate dopamine neurotransmission [44]. The observed increase in tyrosine kinase activity in the dorsal striatum may be linked to enhanced dopaminergic signaling during exercise. This finding aligns with prior studies demonstrating that exercise modulates dopamine pathways, thereby influencing motor activity and motivational states [45–47]. Furthermore, the elevated tyrosine kinase activity may reflect an adaptive response to the increased motor demands and reward mechanisms associated with voluntary exercise.

In the hippocampus, serine/threonine kinase activity plays a critical role in synaptic plasticity, which is essential for learning, memory formation, and cognitive function. Kinases such as CaMKII are implicated in the strengthening of synaptic connections, a process vital for memory enhancement [43]. Our findings of increased serine/threonine kinase activity during VWR suggest that exercise promotes synaptic plasticity, potentially explaining the cognitive benefits of regular physical activity observed in both animal models and humans.

The interplay between genetic, transcriptomic, proteomic, and kinomic factors underlying exercise behavior is highly intricate. Previous studies utilizing polygenic rat models selectively bred for high or low levels of voluntary running have identified molecular pathways involving specific kinases [48]. For instance, overexpression of protein kinase inhibitor alpha in the nucleus accumbens increased running in low voluntary running rats but had no effect on wildtype rats, implicating the cAMP response element-binding protein (CREB) pathway [48]. Similarly, divergent responses to activating protein-1 (AP-1) inhibition were observed, with wildtype rats showing reduced running distances while low voluntary running rats exhibited increased running distances [49]. These findings suggest differences in downstream signaling pathways that contribute to behavioral variability.

Building on prior work, our study demonstrates region-specific kinome changes induced by acute running, particularly in the hippocampus and dorsal striatum. These findings complement earlier research by suggesting that, in addition to differences in downstream signaling, regional variations in protein expression and kinase activity significantly influence voluntary exercise behavior. By integrating these insights, our study contributes to a more nuanced understanding of the molecular mechanisms associated with exercise-related motivation and performance.

### Individual differences in acute exercise behavior and NFkB pathway

Identification of CHUK, IKBKB, and IKBKE as “hits” suggests a deep involvement of the NF-κB signaling pathway in exercise-induced responses. The three catalytic subunits, IKKα (CHUK), IKKβ (IKBKB), and IKKε (IKBKE), work together with the regulatory subunit, IKKγ (NEMO), to form a functional IKK complex. The activation of the IKK complex leads to the phosphorylation and subsequent degradation of IκB proteins, thereby allowing NF-κB to translocate to the nucleus and regulate gene expression [50]. Numerous external stimuli can activate the NF-κB pathway, regulating processes like apoptosis, proliferation, differentiation, and development in a conserved manner [51]. The pathway regulates the pro-inflammatory response initiated by TNFα and IL-1 signaling, which, when chronically activated, contributes to various diseases, including cancer, neurodegeneration, aging, and obesity, suggesting that modulating NF-kB at different levels could mitigate chronic inflammation.

The significant reduction in NF-kB activity in the hippocampus following an episode of voluntary wheel running indicates that acute exercise may have an anti-inflammatory effect in this brain region, reflecting a beneficial impact of exercise and possibly neuroprotection. The moderate negative correlation between running distance and NF-kB activity implies that individuals who engaged in more running exhibited a more pronounced decrease in NF-kB activity. This suggests a dose-response relationship where higher levels of physical activity result in greater reductions in NF-kB activity.

In addition to its role in neuroinflammation, the NF-kB pathway is crucial for regulating synaptic structure and function [52,53]. It influences dendritic spine density and morphology, which are essential for synaptogenesis, synaptic stability, and remodeling [54–57]. NF-kB, as a transcription factor, also regulates the expression of synaptic proteins and receptors, thereby affecting synaptic transmission and efficacy [58,59]. Functionally, NF-kB is involved in synaptic plasticity mechanisms, such as long-term potentiation (LTP) and long-term depression (LTD), which are vital for learning and memory [60,61].

The finding of exercise-induced modulation of NF-κB activity extends beyond the scope of this study, with potential implications for various disease states characterized by dysregulated inflammation. By elucidating the signaling pathways associated with physical activity, our findings provide insights into the development of behavioral interventions aimed at mitigating inflammation and promoting overall health and well-being.

### Potential feedback mechanisms regulating exercise behavior

VWR is an analogous model of the heterogeneity present in voluntary exercise performance [21]. Strengths of the VWR model include that it is natural, rodent-initiated behavior and is highly rewarding to rodents [18,62]. In chronic VWR, running performance improves over time, and it is proposed that running begins as a purposeful, goal-directed behavior motivated by reward circuits [63–66]. Each bout of exercise induces a host of signaling events across widely distributed brain regions, many of which support its beneficial effects [1,12,13]. Perceivably, only a subset of these signaling events acts on relevant brain regions and serves as a mechanism to regulate future exercise behavior itself. Voluntary exercise leads to acute activation of a signaling network potentially containing kinase pathways that feedback through specific striatal circuits to reinforce running behaviors.

### Limitations of this study and Future directions

We used homogenized brain samples, where all the cell types are mixed together. This so-called “blender problem” raises the concern that with all the cells mixed together, cell-subtype-specific changes in protein kinase signaling may not be appreciated [67]. The striatum and hippocampus contain multiple cell types, including excitatory and inhibitory neurons, microglia, astrocytes, oligodendrocytes, as well as endothelial cells. Future work will consider cell subtype specific approaches to delineate changes in kinase signaling pathways and networks associated with voluntary running.

This study focuses on the hippocampus and dorsal striatum, which represent only a subset of the neurocircuitry involved in motivation and reward modulation by exercise. Differential protein expression in other regions, such as the ventral striatum and prefrontal cortex, could provide additional insights into the complex interactions within the mesocorticolimbic circuit that mediate goal-directed behavior.

Another limitation of this work is that we only examined male rats. We plan to include female rats in future studies, as they run significantly more than males and exhibit estrus cycle-dependent fluctuations in running activity [68–72]. Including female rats may reveal additional insights related to estrogen and its effects on physical activity.

## Conclusions

Our study reveals important insights into the molecular changes induced by acute exercise, demonstrating region-specific kinase activity changes and linking these to individual differences in exercise performance. The findings suggest that alterations in kinase signaling, including the NFκB pathway, play a significant role in regulating exercise outcomes. While this research lays a foundation for understanding these complex processes, future studies should explore causal relationships, feedback mechanisms, and the long-term effects of exercise on kinase activity. These insights could guide the development of targeted interventions to enhance physical activity and health.

## Supporting information

S1 File Supplementary information. (DOCX)
