# Supplementary material for: Functional kinome profiling reveals brain protein kinase signaling pathways and gene networks altered by acute voluntary exercise in rats
Source: PLoS One. 2025 Apr 15;20(4):e0321596. doi: 10.1371/journal.pone.0321596 (PMC11999169; doi:10.1371/journal.pone.0321596)
Supplement: S1 File — (DOCX) [file pone.0321596.s001.docx]

**Functional kinome profiling reveals brain protein kinase signaling pathways and gene networks altered by acute voluntary exercise in rats**

***Supplementary Materials***

| *Contents*: |  |
| --- | --- |
| 1. Supplementary Methods | Page 2 |
| 1. Supplementary Figures | Page 11 |
| 1. Supplementary References | Page 13 |

**1. PamGene Kinome Array:**

**1.1. Background**.

The PamGene platform is a well-established and widely cited microarray technology, recognized for its effectiveness in multiplex kinase activity profiling [1-4].

Kinases are essential regulatory proteins involved in various biological processes such as cell cycle control, signal transduction, and gene expression. Profiling kinase activity provides investigators with insights into post-translational modifications, particularly phosphorylation, which govern kinase function and accurately reflect the kinase's functional state within the cell [5]. Often, disruptions in phenotypes may stem from multiple kinases, as several kinases can activate the same downstream target [6]. Therefore, to comprehensively understand the role of kinase networks in a specific biological process, simultaneously screening multiple kinases is an imperative task.

To address this need, the PamStation12 microarray platform has been developed, employing the PamChip4 serine-threonine (STK) and phospho-tyrosine (PTK) microarray chips for efficient screening and analysis.

**1.2. Hardware: PamStation12 and PamChip4**.

The PamGene PamStation12 Kinome Array is a high-throughput peptide array-based platform that enables multiplexed kinase activity profiling and facilitates the unbiased detection of kinase activity via the STK and/or the PTK microarray chips [1-4, 7, 8]. The fundamental design of the two chips is identical, differing only in the specific reporter peptides printed on each.

The PamChip4 STK and PTK chips consist of 144 and 196 reporter peptides, respectively, that are recognized substrates for serine/threonine and tyrosine kinases. Each chip comprises four wells, with approximately 300,000 copies of each peptide in each well. The PamStation12 instrument has the capability of running three chips simultaneously, accommodating up to 12 samples for concurrent analysis on the array platform.

Real-time phosphorylation detection is a key feature of the PamStation12 instrument. This capability allows for the direct measurement of altered kinase activity. For instance, the phosphorylation by the enzyme protein kinase A on the PamChip4 STK chip correlates with its activity in solution [3]. Following several cycles of sample injections and washes, a fluorescent antibody is applied to the PamChip4 PTK chip targeting the phosphorylated residues. In the case of the PamChip4 STK chip, two different antibodies are required to achieve fluorescence. Subsequently, the fluorescence levels can be visualized, with the intensity directly reflecting the extent of reporter peptide phosphorylation, indicative of activity levels.

For a comprehensive overview of the PamGene platform, please refer to Supplementary Figure 1 (S1).

**1.3. Chip Coverage**.

The PamGene instrument offers extensive coverage of the human kinome, facilitating a thorough examination of kinase activity. Out of the approximately 500 kinases constituting the human genome [9, 10], the STK and PTK chips can map 245 out of 376 (65%) serine/threonine kinases and 89 out of 93 (96%) tyrosine kinases, respectively. Additionally, the platform can detect around 18 out of 21 (86%) dual specificity kinases, encompassing approximately 72% of the entire kinome. These results emphasize the efficacy of the PamGene platform in efficiently and accurately identifying altered kinase activity, providing insights into various molecular processes within the studied biological system.

**1.4. Data Generation**.

Data generation for the PamGene platform involves three distinct steps: the Bench step, the Imaging Step, and the Preprocessing step.

During the Bench step, investigators acquire and prepare samples from subjects of interest for analysis on the PamChip4. Standard sample preparation protocols provided by the PamGene Corporation (<https://pamgene.com/ps12/>) ensure result reproducibility and accuracy. In this step, samples undergo treatment with protease and phosphatase inhibitors to control kinase catalytic activity and stability.

Moving to the Imaging step, fluorescent activity images on the chip are generated, utilizing the Evolve 3 kinetic image capture software. Standardized imaging protocols are provided by the PamGene corporation. The PamChip4 PTK chip follows a specific process: Chips are loaded with the processed sample mixture from the Bench step. Blocking buffer and the PamGene reagent mix that includes the fluorescent antibody (allowing for peptide phosphorylation and activity detection) are sequentially added. After a two-minute processing time, images are captured every 5 minutes over 60 minutes at exposure lengths of 5, 25, and 100 milliseconds, allowing real-time recording of reaction kinetics. Standardized imaging protocols, internal control tests, and normalization strategies, provided by PamGene, maintain data sensitivity and reliability, with reported technical variation between chips and runs being <9% and <15%, respectively.

In the preprocessing step, the final stage of data generation, images are processed to quantify the peptide activity via fluorescence levels. The PamGene BioNavigator software is used for this purpose (<https://pamgene.com/wp-content/uploads/2020/09/BioNavigator-User-Manual-vs2.3-2020.pdf>). Annotation data is added to the extracted fluorescence values to identify data at various exposure levels for each sample, ensuring accurate display resolution. A "Signal Background" intensity metric is calculated, accounting for background signal intensity, and a "Signal Saturation" value is measured for each chip well. The final data is displayed in an output table that is subject to further processing by downstream software packages.

Raw signal intensities typically fall within the range of 0 – 3,000. The linear regression slope of signal intensity, averaged across biological replicates, is interpreted as a function of exposure time. This enhances the dynamic range and represents peptide phosphorylation intensity for downstream comparative analyses. The signal intensity ratio between case and control samples is used to calculate fold change (FC) values, with peptides exhibiting a FC of at least 15% (i.e., FC >1.15 or FC <0.85) considered differentially phosphorylated. This threshold aligns with previous reports suggesting that minor changes in kinase activity can trigger biologically relevant alterations [7, 8, 11].

Prior to proceeding to activity analysis, inactive peptides, identified as having a raw signal of ≤5 or an R^2^ of <0.90 during the linear regression, are removed, as values at these thresholds indicate undetectable or non-linear kinase activity in the post-wash phase.

**1.5. Assessment of Upstream Kinases**.

The initial results obtained from the preprocessing step provide information about the phosphorylation status of individual peptides on the chip. Since these outcomes serve as indicators of the peptides or targets that kinases influence, the gathered data represent an indirect measurement of kinase activity. Peptides on the array, and in general, may undergo phosphorylation by multiple kinases. Therefore, pinpointing the individual kinases upstream of a specific peptide becomes essential for a comprehensive understanding of kinase activity.

The process of identifying upstream kinases begins by selecting a specific state of phosphorylation from the input data, typically derived from the final cycle of the array run. For each sample, a variable is defined to summarize the phosphorylation level of that peptide. The change in phosphorylation status within this group is then compared to a control group. Ultimately, a combination of experimental evidence, literature-based information, and computational predictions is employed to identify the most prominent upstream kinases.

Four distinct software packages are available for upstream kinase identification: upstream kinase analysis, kinome random sampling analysis, kinase enrichment analysis v3, and post-translational modification signature enrichment analysis. These packages rely to varying degrees on publicly available mapping databases, and a detailed discussion of each is provided below.

**1.6. Upstream Kinase Analysis (UKA)**.

This software package, developed by the PamGene Corporation (s’-Hertengobosch, Netherlands), incorporates the recommended and standardized protocol for UKA directly into the manufacturer’s BioNavigator methods.

The UKA method relies on a meticulously curated database of kinase-substrate interactions crafted by the PamGene Corporation. UKA processes the raw output from the PamStation12 instrument by filtering low-intensity peptides and scaling/normalizing the entire dataset to ensure that values fall within the 0 – 100 range. Subsequently, the program calculates a “Kinase Score” for each kinase, highlighting those with the highest scores.

The UKA package offers several advantages, including the ability to provide results specific to individual kinases (rather than kinase families) and a notably low false positive rate compared to other software packages. One potential limitation of the UKA package is that its thresholds for specific variables may be too stringent for discovery-based experiments.

**1.7. Kinome Random Sampling Analyzer (KRSA)**.

This package was developed by the Cognitive Disorders Research Laboratory (CDRL) at The University of Toledo College of Medicine and Life Sciences (UTCOMLS), led by Dr. Robert McCullumsmith, M.D., Ph.D. [12].

The algorithm leverages data from the kinome array and mapping from the PamChip4 file. Peptides undergo selective filtering based on defined advancement criteria, considering signal intensity at maximum exposure time and the R^2^ value from the linear regression model. A list of filtered peptides is generated and utilized in subsequent analyses.

Database Curation for Upstream Kinases: KRSA relies on a curated database of upstream kinases associated with peptides on the array. Protein kinases predicted to act on phosphorylation sites within the peptide sequences on the PamChip4 chip are identified using the GPS 3.0 and Kinexus Phosphonet programs [13-15]. Predictions include kinases that may be targeting peptide sequences, ordered by the likelihood of binding. The union of the highest ranked five kinases in Kinexus and kinases with scores more than 2X the prediction threshold in GPS 3.0 are considered predicted kinases for each peptide and are utilized in the KRSA analysis [3].. This list is combined with kinases shown in the literature to act on the phosphorylation sites via PhosphoELM (<http://phospho.elm.eu.org>) and PhosphoSite Plus Plus (<https://www.phosphosite.org>).

Empirical Measures of Statistical Significance: KRSA performs a permutation analysis using the Monte Carlo simulation. First, KRSA takes a random sample of the same number of observed peptides that passed the advancement criteria. For each simulation, upstream kinase counts are accumulated providing a standard normal distribution for the number of times a kinase would be predicted to be assigned by chance. The simulation is run 2,000 times, yielding a distribution histogram as well as median, mean, and standard deviation values for the kinases used in the simulation. The observed (i.e., the number of times a kinase mapped to the peptide from the actual experiment that passed quality control and FC thresholds) and the expected (i.e., from the permutation analysis) kinases are compared. Observed kinases are assigned a z-score, generated based on the number of standard deviations the value is from the mean of the expected upstream kinases. A positive z-score suggests over-involvement (NOT higher activity) of this kinase in the substrate studied versus in the control group. Since the z-score signifies statistical significance (i.e., outside of a pre-determined confidence interval), any kinase with a z-score of >2 is considered a “hit,” warranting further experimental assessment. In some cases, the z-score threshold is adjusted by lowering the stringency to increase the number of candidate kinases for discovery-based studies.

Presentation of Data in KRSA: Heatmaps are generated from signal intensity data based on selected peptides meeting quality control criteria. Linear regression slopes of signal intensity values, representing peptide phosphorylation intensity, are displayed. Violin Plots showcase signal intensity distribution for significant peptides on a per-group basis. Waterfall Plots are generated from z-score values for each kinase, displaying the distribution of z-scores across multiple chips in one assay run.

KRSA complements the PamGene Corporation’s UKA package, employing a less stringent strategy for upstream kinase identification, providing more putative hits for discovery-based studies, where findings may be confirmed with additional studies. A limitation is that KRSA outputs kinase families, not specific kinases. Combining KRSA and subsequent UKA analysis is common, to determine a hit family with KRSA and identify specific family members with UKA or another package, such as KEA3.

**1.8. Kinase Enrichment Analysis 3 (KEA3).**

KEA3, an upstream kinase identification and assignment method, was developed by the Ma’ayan laboratory (<https://maayanlab.cloud/kea3/>). This method relies on known kinase-protein interactions, kinase-substrate interaction data, and associated co-expression and co-occurrence data [16]. The KEA3 web application takes a list of differentially expressed phosphorylated proteins along with their associated FC values as input. Leveraging gene set libraries from kinase-substrate interaction databases, KEA3 generates a ranked list of the top predicted kinases, presenting them as networks, subnetworks, bar graphs, and a cluster-gram.

**1.9. Post-Translational Modification-set Enrichment Analysis (PTM-SEA)**

PTM-SEA is an upstream kinase identification tool developed by the Broad Institute. This is a modification of the Broad Institute’s approach of single-sample Gene Set Enrichment Analysis, which uses a database generated by mass spectrometry of phosphoproteomic data. This method relies on annotated phosphoproteomic sites and known kinase interactions to predict upstream kinases.

**1.10. Integration of Upstream Kinase Assignments Across Packages.**

The mentioned tools employ distinct and independent methods for identifying upstream kinases, highlighting the need for an integration system to determine consensus upstream kinases across datasets. To address this requirement, we employ Creedenzymatic software [19]. The Creedenzymatic program processes outcomes from a minimum of two out of four potential upstream kinase identification analysis methods. It produces a consensus figure with kinases deconvoluted and ranked according to their presence in the results. The integration of these packages enables investigators to pinpoint and select dysregulated kinases for in-depth analysis and subsequent confirmation studies.


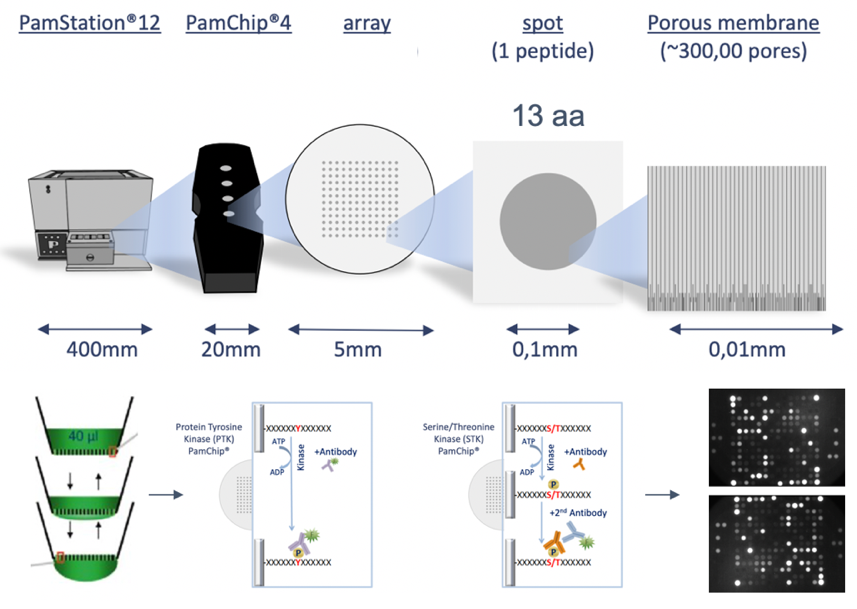
**Supplementary Figures**

**Supplementary Figure S1: The PamGene Platform Workflow**. The PamGene PamStation12 Kinome Array is a high-throughput peptide array-based platform, enabling multiplexed kinase activity profiling and unbiased detection of kinase activity via the serine/threonine kinase (STK) and/or the phosphor-tyrosine kinase (PTK) microarray chips. The PamChip4 PTK chip contains 196 reporter peptides known to be substrates for tyrosine kinases. The PamChip4 STK chip contains 144 reporter peptides known to be substrates for serine/threonine kinases. Each chip contains four wells and each well contains approximately 300,000 copies of each peptide. The PamStation12 instrument can run three chips at once and thus, up to 12 samples may be run simultaneously on the array platform. The PamStation12 instrument detects phosphorylation in real time. After several cycles of sample injections and washes, a fluorescent antibody is applied to the PamChip4 chip against the phosphorylated residues. The fluorescence levels may be visualized, where the intensity of the fluorescence is directly correlated with the extent of reporter peptide phosphorylation (i.e., activity levels). *This figure was reprinted with permission from PamGene International B.V.*

**
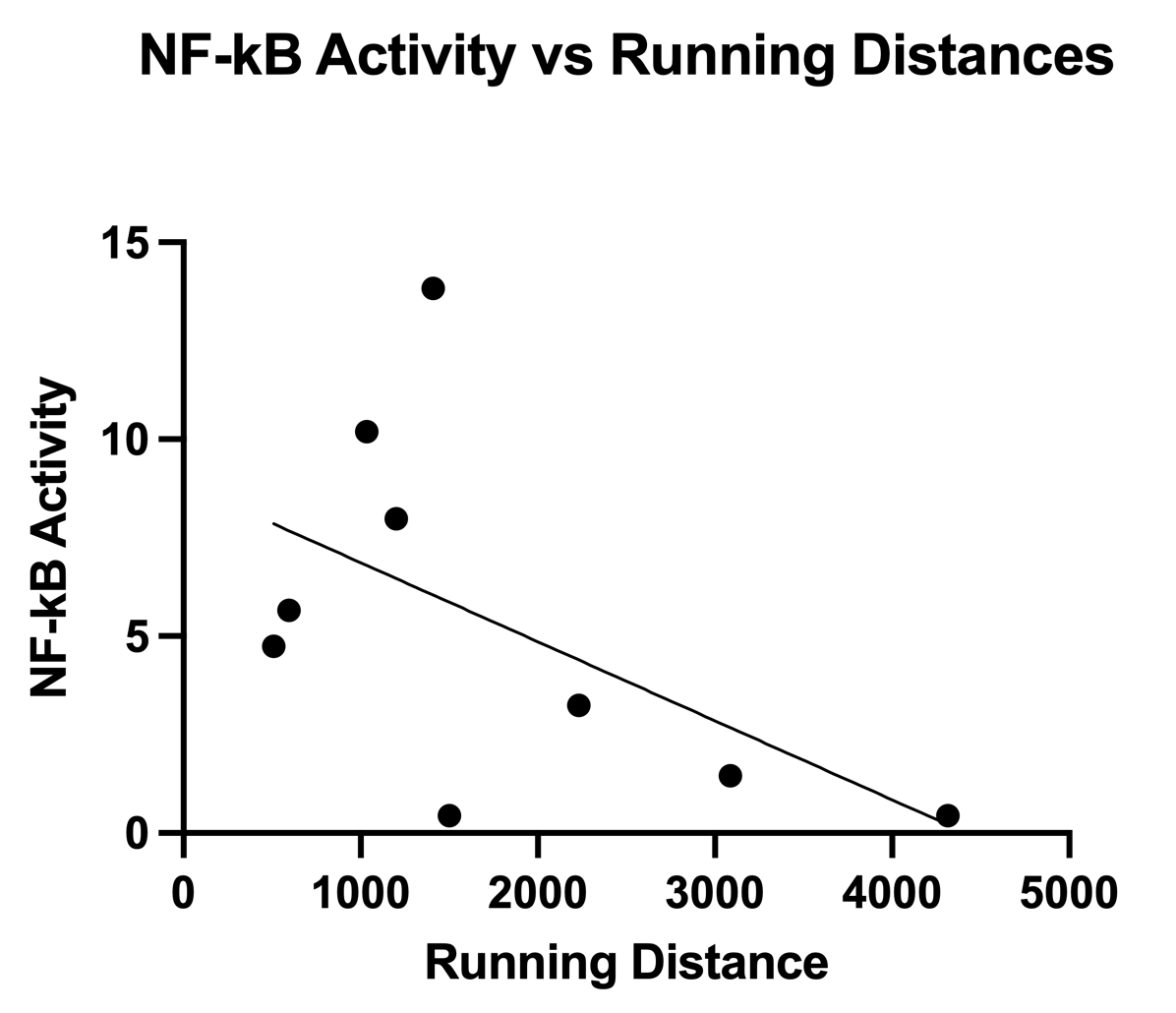
**

**Supplementary Figure S2: Negative correlation between running distance and NF-kB activity in the hippocampus of rats following acute exercise.** Scatter plot of standardized hippocampal NF-kB activity in acute VWR rats. While the data suggest a negative correlation between running distance and hippocampal NF-kB activity, the association did not reach statistical significance (p = 0.131), potentially due to the limited sample size (n=9).

**References**:

[1] R. Arsenault, P. Griebel, and S. Napper, "Peptide arrays for kinome analysis: new opportunities and remaining challenges," *Proteomics,* vol. 11, no. 24, pp. 4595-609, Dec 2011, doi: 10.1002/pmic.201100296.

[2] A. Baharani, B. Trost, A. Kusalik, and S. Napper, "Technological advances for interrogating the human kinome," *Biochemical Society Transactions,* vol. 45, no. 1, pp. 65-77, 2017/02/08/ 2017, doi: 10.1042/BST20160163.

[3] E. Bentea *et al.*, "Kinase network dysregulation in a human induced pluripotent stem cell model of DISC1 schizophrenia," *Mol Omics,* vol. 15, no. 3, pp. 173-188, Jun 10 2019, doi: 10.1039/c8mo00173a.

[4] R. Hilhorst, L. Houkes, A. van den Berg, and R. Ruijtenbeek, "Peptide microarrays for detailed, high-throughput substrate identification, kinetic characterization, and inhibition studies on protein kinase A," *Analytical Biochemistry,* vol. 387, no. 2, pp. 150-161, 2009/04/15/ 2009, doi: 10.1016/j.ab.2009.01.022.

[5] M. Mann and O. N. Jensen, "Proteomic analysis of post-translational modifications," (in eng), *Nat Biotechnol,* vol. 21, no. 3, pp. 255-61, Mar 2003, doi: 10.1038/nbt0303-255.

[6] J. Zhang, P. L. Yang, and N. S. Gray, "Targeting cancer with small molecule kinase inhibitors," (in eng), *Nat Rev Cancer,* vol. 9, no. 1, pp. 28-39, Jan 2009, doi: 10.1038/nrc2559.

[7] J. L. McGuire *et al.*, "Abnormalities of signal transduction networks in chronic schizophrenia," *NPJ Schizophr,* vol. 3, no. 1, p. 30, Sep 12 2017, doi: 10.1038/s41537-017-0032-6.

[8] C. R. Dorsett *et al.*, "Traumatic Brain Injury Induces Alterations in Cortical Glutamate Uptake without a Reduction in Glutamate Transporter-1 Protein Expression," *J Neurotrauma,* vol. 34, no. 1, pp. 220-234, Jan 1 2017, doi: 10.1089/neu.2015.4372.

[9] G. Manning, D. B. Whyte, R. Martinez, T. Hunter, and S. Sudarsanam, "The protein kinase complement of the human genome," *Science,* vol. 298, no. 5600, pp. 1912-1934, 2002/12/06/ 2002, doi: 10.1126/science.1075762.

[10] G. Manning, "Genomic overview of protein kinases," *WormBook : the online review of C. elegans biology,* pp. 1-19, 2005 2005, doi: 10.1895/wormbook.1.60.1.

[11] J. A. Appuhamy, W. A. Nayananjalie, E. M. England, D. E. Gerrard, R. M. Akers, and M. D. Hanigan, "Effects of AMP-activated protein kinase (AMPK) signaling and essential amino acids on mammalian target of rapamycin (mTOR) signaling and protein synthesis rates in mammary cells," *J Dairy Sci,* vol. 97, no. 1, pp. 419-29, 2014, doi: 10.3168/jds.2013-7189.

[12] E. A. K. DePasquale *et al.*, "KRSA: An R package and R Shiny web application for an end-to-end upstream kinase analysis of kinome array data," (in en), *PLOS ONE,* vol. 16, no. 12, p. e0260440, 2021/12/17/ 2021, doi: 10.1371/journal.pone.0260440.

[13] Y. Xue *et al.*, "GPS 2.1: enhanced prediction of kinase-specific phosphorylation sites with an algorithm of motif length selection," (in en), *Protein Engineering Design and Selection,* vol. 24, no. 3, pp. 255-260, 2011/03/01/ 2011, doi: 10.1093/protein/gzq094.

[14] C. Wang *et al.*, "GPS 5.0: An Update on the Prediction of Kinase-specific Phosphorylation Sites in Proteins," (in eng), *Genomics, Proteomics & Bioinformatics,* vol. 18, no. 1, pp. 72-80, 2020/02// 2020, doi: 10.1016/j.gpb.2020.01.001.

[15] Y. Xue, F. Zhou, M. Zhu, K. Ahmed, G. Chen, and X. Yao, "GPS: a comprehensive www server for phosphorylation sites prediction," *Nucleic Acids Research,* vol. 33, no. suppl_2, pp. W184-W187, 2005/07/01/ 2005, doi: 10.1093/nar/gki393.

[16] M. V. Kuleshov *et al.*, "KEA3: improved kinase enrichment analysis via data integration," *Nucleic Acids Res,* vol. 49, no. W1, pp. W304-W316, Jul 2 2021, doi: 10.1093/nar/gkab359.

[17] K. Krug *et al.*, "A Curated Resource for Phosphosite-specific Signature Analysis," *Mol Cell Proteomics,* vol. 18, no. 3, pp. 576-593, Mar 2019, doi: 10.1074/mcp.TIR118.000943.

[18] J. Cox and M. Mann, "MaxQuant enables high peptide identification rates, individualized p.p.b.-range mass accuracies and proteome-wide protein quantification," (in eng), *Nat Biotechnol,* vol. 26, no. 12, pp. 1367-72, Dec 2008, doi: 10.1038/nbt.1511.

[19] *CogDisResLab/creedenzymatic: v 5.0.0 Version Reset*. (2022). Zenodo. Accessed: 2022/08/31/20:19:53. [Online]. Available: [https://zenodo.org/record/6363767[Online](https://zenodo.org/record/6363767%5bOnline)]. Available: <https://zenodo.org/record/6363767#.Yw-_gezMLzc>
